# Beyond the Meeting: Patient and Public Involvement Practices and Barriers to Inclusive Participation in Stroke Research

**DOI:** 10.1111/hex.70903

**Published:** 2026-10-05

**Authors:** David Wyatt, Sophie Rowland‐Coomber, Eleanor Stevens, Christopher McKevitt, Madeline Cruice, Timothy Neate, Eva S. Emmett, Matthew D. L. O'Connell, Charles Wolfe, Iain J. Marshall

**Affiliations:** ^1^ Department of Society and Politics Aston University Birmingham UK; ^2^ School of Life Course and Population Health Sciences King's College London London UK; ^3^ Department of Primary Care and Data Science King's College London London UK; ^4^ Department of Allied Health, City St George's University of London London UK; ^5^ Department of Informatics King's College London London UK; ^6^ NIHR Applied Research Collaboration (ARC) South London at King's College Hospital NHS Foundation Trust London UK

**Keywords:** health inequalities, inclusive research, infrastructure, participation, Patient and Public Involvement, qualitative research, stroke

## Abstract

**Background:**

Patient and Public Involvement (PPI) aims to embed patient perspectives in all aspects of health research. In stroke, where diverse impairments affect communication, cognition, and mobility, inclusive PPI is particularly critical yet challenging. Little is known about how stroke PPI is currently practised or what barriers researchers face in supporting the participation of stroke survivors with complex needs.

**Objectives:**

To understand how stroke PPI is currently structured and practised, what resources it requires, how research teams support stroke survivors with diverse impairments and what barriers exist to more inclusive practice.

**Methods:**

We conducted a national questionnaire with Principal Investigators of NIHR, UKRI and Stroke Association‐funded stroke research projects active between October 2019 and January 2025 (31 responses representing 104/185 eligible projects). Open‐ended questions elicited detailed accounts of PPI practices, resource requirements, adaptations to support diverse participation and barriers to inclusion. Data were analysed using qualitative description and thematic analysis, drawing on concepts of infrastructure to examine how the way PPI is organised shapes participation.

**Results:**

Four interconnected themes were identified. Despite considerable variation in scale and sophistication, the group meeting dominated PPI practice, functioning as taken‐for‐granted infrastructure through which involvement happens. Resource constraints, particularly unfunded coordination time and absent specialist communication support, limited what was viable. Researchers consistently reported lacking expertise to support stroke survivors with severe impairments, relying heavily on carers whose involvement sometimes functioned as proxy representation. While participants demonstrated creativity and commitment, PPI groups remained predominantly ‘white, middle class, and [with] minor stroke severity’, with innovation not leading to more inclusive participation.

**Discussion:**

Group meetings dominate stroke PPI, but they systematically exclude stroke survivors whose impairments make participating in meetings difficult. Achieving inclusive stroke PPI requires not merely adapting meetings but reimagining participation infrastructure, adequately resourcing diversity work and addressing expertise gaps through multidisciplinary support.

**Lived Experience or Public Contribution:**

This research was informed by prior workshops with stroke survivors and carers from two PPI groups, who identified barriers to PPI participation and emphasised the need for methods to support more severely affected stroke survivors. Stroke survivors shaped questionnaire content and helped refine the findings and themes.

## 1 Introduction

Patient and Public Involvement (PPI), defined as ‘research being carried out “with” or “by” members of the public rather than “to”, “about” or “for” them’, has become fundamental to health research in the UK and internationally [1]. The rationale for PPI encompasses normative arguments (patients' right to involvement), consequentialist perspectives (improving research quality and relevance), and political dimensions [2]. When practised effectively, PPI has the potential to embed patient and public perspectives throughout the research process, ensuring that research addresses questions that matter to those most affected, is designed in ways that are feasible and acceptable to patients, and leads to outcomes that align with their self‐identified needs and priorities [3, 4, 5]. Whether PPI can deliver on this potential, however, depends not only on how open researchers are to PPI input, but on who participates, how, and how that participation is facilitated.

Stroke is an important case through which to examine inclusion in PPI. As the single largest cause of complex disability in adults [6], stroke impacts individuals heterogeneously. Stroke survivors may experience communication difficulties (affecting approximately two‐thirds of stroke survivors [7]), cognitive impairments, vision problems, fatigue, reduced mobility and mental health challenges [8, 9, 10]. While PPI plays a key role in stroke research design, as well as improving services and patient outcomes [11, 12], the variation in stroke experience and impact presents practical challenges for how we involve stroke survivors with diverse experiences in research.

Recent work on engaging underserved communities, particularly in terms of black and ethnic minority groups, acknowledges that ‘methods traditionally employed in [patient and public involvement and engagement] can lead to the exclusion of some communities and the exacerbation of existing inequalities' [13, p. 1], highlighting the importance of attending to not only who participates in PPI but also how PPI is facilitated. In this paper, we operationalise ‘underserved’ in line with NIHR INCLUDE guidance [14]. While acknowledging the importance of demographic characteristics in experiences of illness and exclusion, we focus on the often‐overlapping issue of the severity of stroke. For stroke PPI, this means attending to how the severity of stroke‐related impairment shapes who can participate, recognising impairment as a key axis of exclusion alongside demographic patterns. For example, researchers have identified barriers to stroke survivor participation due to stroke‐related communication difficulties [15, 16]. When teams adjust procedures and draw on specialist expertise, particularly speech and language therapists (SLTs), however, stroke survivors with communication difficulties were able to participate fully [17, 18].

The ‘PPI meeting’ as the primary mechanism of doing PPI has received little critical attention. Meetings, whether face‐to‐face or online (the latter, initially, a response to the COVID‐19 pandemic), privilege particular modes of participation, namely, synchronous attendance, real‐time communication, sustained concentration, and comfort in group settings. For stroke survivors with severe communication difficulties, cognitive impairments, fatigue, or mobility limitations, meetings may be a barrier to participation. Critical scholarship on PPI practice draws our attention to what is at stake when involvement formats go unquestioned. Komporozos‐Athanasiou et al. [19] show how routinised practices can function as ‘political ritual’, negating transformative potential, while Mosse [20] highlights how formal participation structures ‘disembed’ lay knowledge from social context, filtering out embodied and relational ways of knowing. Together, these critiques suggest that when meeting formats become the unquestioned default, PPI risks both routinising participation and excluding the very knowledge it claims to seek.

Group‐based, synchronous, event‐structured formats, whether termed meetings, workshops, advisory boards, or focus groups, appear to be a persistent component of PPI across health conditions more broadly. Pollock et al.'s [21] review of stakeholder involvement in systematic reviews found that, amongst the minority of reviews with sufficient detail to characterise PPI methods, face‐to‐face was the dominant format (81%), taking the form of group meetings (53%), larger workshops/public events (19%) or both (9%). Similarly, in cancer and psychiatric research, group‐based formats (workshops, focus groups, advisory boards) feature alongside one‐to‐one interviews as commonly applied PPI methods [22, 23], suggesting that while individual‐level formats are also used elsewhere, group‐based involvement remains widespread.

A related but distinct critique concerns how the value of involvement is assessed. Russell et al. [24] argue that the emphasis on measuring PPI impact has led to an instrumental view of PPI as an ‘intervention’ with quantifiable effects on recruitment and research quality. This focus on measurement favours what is easy to count (numbers of people, meetings held) over what matters most about the practices of doing PPI (e.g., meaningful dialogue, power‐sharing, whose voices are heard). Karlsson et al.'s [25] review found that researchers typically remain decision‐makers throughout research, with public contributors' input representing ‘filtered lived experiences, tailored to meet dominant hermeneutical frameworks and adjusted to be compatible with existing research structures’.

To understand how these structural and conceptual challenges appear in stroke PPI practice, we conducted a national questionnaire with Principal Investigators of National Institute for Health and Care Research (NIHR), UK Research and Innovation (UKRI) and Stroke Association‐funded stroke research projects that were active between October 2019 and January 2025. Through open‐ended questions, we sought to understand: (1) How is PPI currently structured and practised in stroke research? (2) What resources do PPI activities require? (3) How do research teams currently attempt to support the involvement of stroke survivors with more severe impairments (referred to as severe stroke here)? (4) What barriers do researchers identify to more inclusive PPI practices?

We draw on accounts of ‘infrastructure’, a concept that became central during analysis (see Methods), as an analytic lens. Star and Ruhleder [26] argue that infrastructures are characterised by several key properties. They are embedded within other structures and social arrangements. They are learned as part of membership in communities of practice. Crucially, they typically become visible only when they break down [27]. Adopting infrastructure as an analytic lens allows us to see seemingly cultural barriers (e.g. expectations about participation style, meeting norms) as actually embedded in material and organisational arrangements (funding structures, institutional timelines, payment systems). It also foregrounds the stabilised practices that make certain forms of PPI possible while rendering alternatives difficult to imagine. Importantly, it shifts analytical attention from individual failures or differences to systemic patterns. In our analysis, we attend to both the formal meeting infrastructure and the substantial adaptive work required to maintain it—the ‘articulation work’, namely the often‐invisible labour of making systems work in practice [28, 29]. Papoulias and Brady [30] demonstrate how PPI coordinators' invisible or behind‐the‐scenes labour remains systematically undervalued even as they are held accountable for delivering ‘transformational’ involvement. An attention to infrastructure facilitates a shift in analytical focus from asking why certain stroke survivors do not participate, towards questioning what arrangements make their participation difficult to envisage.

Through qualitative data from researchers, we illuminate how well‐intentioned, thoughtful and creative practices may reproduce patterns of exclusion, identifying structural, resource, and knowledge barriers that must be addressed. While focused on stroke, the insights have broader relevance for PPI in health conditions characterised by complex needs.

## 2 Methods

### 2.1 Sampling and Recruitment

We identified Principal Investigators of stroke research projects funded by the NIHR, UKRI, or the Stroke Association that were active between October 2019 and January 2025. Projects were identified through these funders' publicly available funding databases. We included projects where stroke or stroke‐related impairments were the sole focus and required ethics approval. In practice, this meant we excluded quality improvement projects and studies where stroke was one of multiple conditions examined. This yielded 185 eligible projects.

### 2.2 Data Collection

Data were collected via online questionnaire, using Qualtrics, emailed to the Principal Investigators of the 185 eligible studies in January 2025 (see Supporting File S1). Members of our PPI group reviewed the questionnaire prior to distribution, and their input shaped several questions, including a stronger focus on the costs of PPI activity and on how researchers engage with participants (e.g., what kinds of input are sought). The questionnaire consisted of open‐ended questions designed to elicit detailed responses about their PPI group and PPI activities, resource requirements, adaptations to support those with severe stroke, and ideas for improving equity and inclusiveness. This approach allowed participants to describe their PPI practices in their own terms, capturing nuanced, context‐specific knowledge. Participants were asked to indicate which eligible study or studies their responses related to, allowing both individual and group responses. The questionnaire remained open for 8 weeks. Two reminder emails were sent during this time.

### 2.3 Data Analysis

We analysed responses using qualitative description [31], appropriate for research seeking to provide comprehensive summaries of phenomena whilst staying close to participants' own language, and thematic analysis [32]. Our approach was inductive. Coding, led by a sociologist (DW), was initially descriptive and progressively became more interpretive as codes were grouped into themes. Codes and themes were refined through discussion with co‐author SRC and later the wider, multidisciplinary research team who authored this paper, including a member with lived experience of stroke. Themes were also refined further following discussion with members of our PPI group.

During the analysis process, we drew on the concept of infrastructure as an analytic lens to explore how the organisation of PPI shapes participation, attending particularly to what becomes taken‐for‐granted, what requires maintenance work, and where infrastructure becomes visible through breakdown or, in the context of our work, through questioning.

### 2.4 Reflexivity

This study involved researchers asking other researchers about PPI practices. Participants may have felt inclined to present work favourably, given that PPI is increasingly scrutinised by funders. We mitigated this through invitation framing that emphasised our interest in challenges, and through open‐ended questions, inviting reflection. As researchers ourselves engaged in PPI work, we approached analysis recognising that challenges identified are generally systemic rather than individual failings.

## 3 Results

Invites were sent to 154 principal investigators across 185 research projects. We received 31 complete responses representing experiences from 104 distinct stroke research projects, a project‐level response rate of 56.2% (104/185). Participants held diverse roles (e.g. principal investigators, co‐investigators, researchers, PPI leads, supervisors) and represented universities and NHS across Great Britain (see Table 1), many responding on behalf of multiple projects, ranging from one to 23. Some participants also reported having multiple roles on projects (e.g., acting as both principal investigator and PPI lead). Therefore, the role counts in Table 1 total more than 31. Of the 31 participants, 21 identified as principal investigators (8 solely as principal investigators), 10 as PPI lead, 9 as co‐investigator, 4 as co‐principal investigator, 3 as supervisor, 3 as mentor, 2 as trial manager, 2 as researcher, and 1 as sitting on a Project Oversight Committee. One participant did not report a specific role, though independent grant records confirm they held a principal investigator position on at least one externally funded stroke research project.

**Table 1:** Participant roles and number of projects represented.

Participant	Principal Investigator	Co‐Principal Investigator	Co‐Investigator	Patient and Public Involvement Lead	Researcher	Supervisor	Mentor	Trial Manager	Project Oversight Committee	Grants reported (*n*)
Participant 1	X		X	X						2
Participant 2	X					X	X			8
Participant 3	X	X	X							4
Participant 4	X			X						1
Participant 5				X	X					1
Participant 6	X									2
Participant 7				X				X		4
Participant 8	X									2
Participant 9				X						2
Participant 10										0*
Participant 11	X									1
Participant 12	X	X	X							4
Participant 13		X								1
Participant 14	X			X						2
Participant 15	X		X							3
Participant 16								X		1
Participant 17				X						1
Participant 18	X					X				4
Participant 19				X						1
Participant 20	X		X				X		X	4
Participant 21	X									1
Participant 22	X									1
Participant 23	X		X	X						4
Participant 24	X									3
Participant 25	X		X							3
Participant 26				X						1
Participant 27	X									2
Participant 28	X	X	X				X			23
Participant 29	X									1
Participant 30	X		X			X				15
Participant 31					X					2
Total	**21**	**4**	**9**	**10**	**2**	**3**	**3**	**2**	**1**	**104**

* Principal Investigator on at least one grant, but not self‐reported in questionnaire.

Four themes were identified: (1) the meeting as the dominant infrastructure; (2) resource constraints as barriers to inclusivity; (3) limited expertise in supporting complex needs; and (4) innovation for inclusion and its limits. These themes, while analytically distinct, were interconnected in participants' accounts, reflecting the complex, multi‐layered nature of the barriers to inclusive PPI.

### 3.1 The Meeting as the Dominant Infrastructure

Among the 31 responses, almost all PPI involvement was framed through the language and structure of the group meeting either directly using ‘meeting’ (81%) or through inferring meeting‐based activities (e.g., ‘the group meet quarterly, face‐to‐face’) (further 10%). This apparent uniformity masked considerable diversity in what happened within meetings and the substantial labour required to make them work. Participants described meetings varying in scale, purpose, and organisation:

[C]ore group of 10 stroke survivors and 2 carers + ad hoc additional members for specific projects aged between 40 and 80 core group meets in person, some project groups meet online the PPI group is specific to our research group and not used by other researchers than our group members and students in our group—input on design of new studies, design and management of current ongoing studies, input on ethics, decisions of participant facing materials, core outcome measures on QoL, dissemination plans + dissemination help.

(Participant 2)

The breadth of activities (from study design through to dissemination) demonstrates that ‘the meeting’ encompasses a broad set of practices. Meetings served to support the review of grant applications, co‐design interventions, discuss findings, develop participant‐facing materials and shape dissemination strategies. They varied in duration, frequency, modality (face‐to‐face, online, hybrid), and leadership (researcher‐led vs. stroke survivor‐chaired).

This diversity extended to efforts to engage underserved communities in general:

My initial group had 6–7 attendees all were minor strokes. We met online, 4 times over a year. The group were involved in coming up with the research question, input into the design of the complex intervention, input into research design, underpinning theory and outcome measures used. I have met with underserved communities (refugee women) to gain an understanding of their views on health and recovery and engagement in research. I have attended community health events in communities with ethnic diversity to understand their priorities and views on health. I have met with people in the community as a group of 2 and on a 1 to 1 basis when requested.

(Participant 9)

Here, ‘meetings’ span formal hospital/university‐based PPI groups, community health events, and paired conversations. Yet across this diversity, meetings remained the infrastructure, the taken‐for‐granted organisational form through which all this activity happened. One‐to‐one consultations, community events, and paired conversations were positioned as supplements to, rather than alternatives to or central components of meeting‐based PPI. In this uniformity of framing, rather than uniform practice, the meeting functions as the discursive and organisational infrastructure through which PPI is imagined and enacted. It becomes limiting, however, when the PPI meeting functions as the *only* framework, a framework adopted without critical thought about why this approach is most appropriate and where alternatives are rarely considered or imagined. In stroke, some of a PPI meeting's particular requirements (synchronous attendance, real‐time interaction, scheduled events, group participation) become invisible barriers to the many stroke survivors for whom participating in involvement work on these terms would prove challenging. When this normative infrastructure dominates, those stroke survivors whose stroke‐related impacts make meeting participation difficult become systematically excluded from shaping research, regardless of their knowledge, experience, or desire to contribute.

### 3.2 Resource Constraints as Barriers to Inclusivity

The meeting may be the taken‐for‐granted organisational form of PPI, but it is far from an easy option. Resources and resource constraints were consistently raised by participants, shaping what PPI activity was viable. Time was the most frequently mentioned resource requirement, cited by 24 of the 31 participants. Accounts documented the substantial labour needed to complete PPI and, by extension, maintain the PPI meeting infrastructure. As Star and Strauss [28] observe, infrastructures require ongoing maintenance work to function, yet this work often remains invisible and undervalued. In stroke PPI, the mismatch between what meeting infrastructure requires and what gets resourced reveals what is valued and what is not. The most detailed accounts of this labour came from participants in PPI lead roles, who were closest to its day‐to‐day coordination. One participant articulated the breadth of this time commitment:

[W]e have found that it takes approx. half a day a week to support PPI activity (including supporting PPI co‐applicant/PPI group chair; arranging transport; arranging finances; supporting access to the building; arranging food; supporting online access; providing materials in an accessible format; engaging with group members outside of the group and meeting with them on a 1:1 basis to maximise their ability to contribute in a group setting; supporting attendance and presentations at conferences). Payment‐ as per NIHR guidance; contribution towards broadband—as per NIHR guidance; transport costs for PPI members and staff to travel to meet with PPI members; refreshments (typically approx. £10 a head for half day meetings).

(Participant 7)

The labour described spans logistics, administrative coordination, technological support, accessibility work, and ongoing relationship maintenance, namely ‘articulation work’ [28, 29]. The work happening within meetings is only a small part of this. Relationships must be built and nurtured over time. Infrastructure studies emphasise that infrastructure is never simply ‘there’, it requires constant maintenance work to remain functional [28, 33]. This maintenance work was particularly acute when attempting to build diverse PPI groups:

Currently we are undertaking a large project to try to increase diversity in PPI reps‐this is taking considerable time and money including time outside of the working week as some events happen at a weekend.

(Participant 8)

Participant 8 highlights that efforts to promote diverse participation in PPI sit outside of typical working patterns, often becoming additional labour that is unfunded or inadequately resourced.

Several participants emphasised the relational dimension of PPI work. Building trust could not be rushed:

Planning a PPI group, liaising with attendees and researchers' takes considerable time. I also send out emails afterwards thanking attendees for coming to the group and with a reminder about the next group. I send an email about a month before the group and then another a week before. Getting feedback from researchers on the impact on their research of the session is very challenging and they often don't send this despite frequent requests. Organising payment for transport is also time consuming and slow. We have to pay for tea and coffee which currently comes out of a research budget. I book the room a year in advance now.

(Participant 9)

From advance planning and ongoing communication to difficulty paying for transport, this account reveals friction between PPI requirements and institutional systems not designed to support them. This articulation work is rendered visible through participants' accounts. With this volume of activity, it is unsurprising that multiple participants explicitly named the need for dedicated PPI roles:

A dedicated PPI lead within a study is essential to ensure appropriate time is allowed to facilitate meaningful PPI engagement. Due to the individual needs of stroke survivors, it can take a lot of time to make arrangement for any in person meetings in particular. Also, an understanding of cognitive effects of stroke as well as potential fatigue is important. Adequate PPI funds are essential in all research activities, restrictions on funding can make this difficult. Payment of PPI time, PA[personal assistant]/carer time to support their involvement, refreshments and room hire. On average for any project grant I would try and budget between 5–10k.

(Participant 1)

The emphasis on ‘individual needs’ acknowledges that stroke survivors are not a homogeneous group. The suggested budget of £5‐10,000 per project signals that meaningful PPI represents substantial resource allocation. Notably, this includes ‘PA/carer time to support their involvement’, recognising that for some stroke survivors, participation depends on paid support. While many mentioned adapting materials or including carers, participants identified a funding gap for wider professional support to enable participation by those with complex communication needs (such as speech and language therapists).

Across these responses, resources emerge as more than practical constraints—they express what gets valued. When PPI coordination time goes uncosted or underestimated, when professional communication support remains unfunded, certain forms of participation are resourced while others are not.

### 3.3 Limited Expertise in Supporting Complex Needs

While participants described various adaptations to support those with severe stroke, a lack of expertise and confidence in facilitating PPI with those with more severe or complex needs was consistently acknowledged. Such adaptations may also benefit older stroke survivors who frequently have physical or cognitive impairments unrelated to stroke. These well‐intentioned strategies revealed as much about the limits of current practice as about its possibilities.

The most common adaptation was involving carers, mentioned by 13 participants. As one noted: ‘2 of our group have aphasia, many have cognitive impairments. inclusion of carer + slowing down, ensuring sufficient time, small group facilitation’ (Participant 2). These responses position carers as essential facilitators.

Many also described adaptations to materials and formats. One participant explained:

All written information is provided in advance of the meetings. Audio recordings are provided to accompany PowerPoint presentations. Members can provide feedback in ways that are easiest for them—via email, verbally during or after the meeting, using the chat function on MS Teams, via text message during and after the meeting … Being flexible on how you engage with members—one member wanted to be involved but found attending the MS teams meeting challenging—we visit them at home but after watching a clip of the meeting they feel more confident to join future teams meetings.

(Participant 24)

Some participants demonstrated creative thinking about where PPI might happen:

We have attended a communications support group to gain a better understanding of those who have communication impairment. This has the benefit of people feeling at ease, as in a group that is familiar to them, often the attendees have support from people who attend with them/the group organisers. Sometimes it is important to go out to see people rather than expecting them to attend a group. This also facilitates F2F discussion. I think this requires researchers to think who needs to be consulted and find ways to do this that will be effective and acceptable to those with whom they want to consult.

(Participant 8)

Others were more candid about the limits of their current PPI work:

We do not have any attendees with severe stroke‐related impairments. Only two attendees walk with a stick and have obvious ongoing physical impairments from their stroke. We would like to have people attending with more severe stroke‐related impairments. My initial thoughts on the barriers to this is transport if F2F because hospital transport is not an option because of timing and my second concern is how to support the person with toileting if needed. We do not have any attendees with severe cognitive or language impairments. I think that involvement for these people would need to be in smaller groups with more support provided.

(Participant 9)

One participant highlighted how the research focus itself shapes who participates:

I've found it difficult to engage people who are not mobile in PPI events, but this may also be due to the nature of my research which has not been focused on non‐ambulant stroke survivors.

(Participant 14)

Participant 14's account exposes how a research topic can determine whose participation they seek, reinforcing agendas that overlook particular, and more diverse, stroke experiences, reducing stroke survivors to their (lack of) specific impairments.

The nature of carer involvement often remained ambiguous, however. The dual positioning of carers as supporters of stroke survivor participation and as stakeholders raised questions about whose voice was ultimately heard, particularly when those with severe stroke were absent from PPI groups. Widespread carer involvement, although often a practical necessity, risks carers potentially functioning as a proxy for those with severe impairments rather than contributing alongside them.

Yet when involving those with severe communication impairments, significant mobility limitations, complex cognitive needs or substantial post‐stroke fatigue, many expressed uncertainties, relied heavily on carers, or simply acknowledged these individuals were underrepresented. The gap was not one of intention but of expertise, resources, and established practice. For example, little mention was made of aids to support those with communication difficulties (beyond producing aphasia friendly materials distributed in advance) or cognitive difficulties. But, as one participant summarised, this is not a job for a single person: ‘Much like stroke care [inclusive PPI] needs a multidisciplinary team approach drawing skills from speech and language therapy, occupational therapy and neuropsychology. All with the aim of inclusion and accessibility’ (Participant 4). This call for multidisciplinary teams acknowledges that current infrastructure lacks the expertise to support diverse participation, suggesting that more inclusive PPI requires not just adapted meetings but differently structured involvement altogether.

### 3.4 Innovation for Inclusion and Its Limits

Despite acknowledged expertise gaps, when asked about potential ways we could make stroke PPI more inclusive of those with more severe stroke, participants described innovative practices:

Ask people what we can do to help them. Make it clear that we can provide support. Reassure people that there are no wrong answers and that their opinions are highly valued, even where they think they might be ‘off‐target’ (a quote from one of our PPI members who had cognitive difficulties). Organise activities that can be accessed by people with different problems.

(Participant 12)

The direct quote (‘off‐target’) from a PPI member with cognitive difficulties illustrates how self‐doubt can inhibit participation, while the reassurance from the research team can help alleviate anxiety.

One participant suggested comprehensive principles including accessible communication, multiple participation modes, payment fostering equality ‘with senior clinicians/HCPs and researchers’, acting on feedback, and crucially: ‘allowing people to work together with you instead of work for you’ (Participant 26). This distinction between working ‘with’ versus ‘for’ signals awareness that inclusion requires partnership, not merely accommodation.

Another emphasised adaptive responsiveness: ‘Remaining flexible and responding to individual needs as they change or develop is also important’ (Participant 5), acknowledging stroke recovery's dynamic nature. Several stressed sustained community engagement: ‘Engage pro‐actively on a sustained regular basis with stroke groups, community groups etc’ (Participant 20), challenging project‐by‐project approaches.

The need for flexibility in approach, as noted by one participant, extends beyond format:

We need to be flexible in how PPI is delivered and accept that group meetings will not work for everyone. Some of my participants I have met on a one‐to‐one basis because they have said that they have not disclosed their stroke to anyone and therefore would not attend a group. We need to be willing to go to people in the community, this may involve community groups where not everyone has experience of stroke. We need to consider some degree of matching of attendees so that severely disabled participants are not in a group of minor strokes or vice versa. We need to understand and consider cultural norms and practices and accommodate these. We also need to consider a person's level of health literacy.

(Participant 9)

This reveals exclusion operating beyond impairment (e.g., non‐disclosure, group discomfort, cultural norms). The suggestion of ‘matching of attendees’ by severity acknowledges that mixing vastly different experiences may privilege some voices while marginalising others.

Yet despite this sophisticated understanding and creative adaptation, participants consistently acknowledged limited success in achieving diversity. One stated directly:

How we identify the PPI members currently is very swayed towards white middle class, and minor stroke severity. Funds to build longer‐standing relationships with wider community groups would be helpful in increasing diversity.

(Participant 1)

This observation (‘white middle class, and minor stroke severity’) names three axes of exclusion operating simultaneously, framing diversity as requiring sustained investment rather than simply recruitment fixes. It is noteworthy in the data that there was also no mention of those who do not speak English or speak English as a second language.

Another elaborated on the long‐term process of facilitating diversity in PPI:

I think gaining a diverse PPI group is difficult … this is a long‐term project and there is a small group of staff seeking to do this on behalf of the larger community of stroke researchers … it takes considerable time to build trusted and sustained relationships with people that would not usually get involved with PPI activities … it will take months, probably years.

(Participant 8)

The recognition that success requires ‘months, probably years’ underscores that diversity cannot be achieved within typical project timescales.

Even deliberate work going out into the community yielded limited results. Despite identifying members ‘from community groups which are not stroke‐specific to increase diversity (e.g., in particular ethnic communities)’, one participant noted they ‘achieved little ethnic diversity’ (Participant 12). Demographic patterns repeated across accounts regardless of group size, suggesting that scale alone does not confer diversity and structural barriers extend beyond individual effort.

While participant accounts demonstrate remarkable creativity and commitment to inclusion, their PPI groups remained demographically narrow and often limited to stroke survivors living with less severe ongoing impacts of stroke. Innovation in how PPI happened (accessible materials, flexible formats, community outreach) had not fundamentally changed who participated. The infrastructure of the meeting, the resource constraints detailed earlier, and the expertise gaps combined to create systematic exclusion that good intentions and creative adaptation could not overcome.

## 4 Discussion

The participants in our study demonstrated considerable care, creativity and commitment to including stroke survivors' input in research. Yet despite this labour, the structural dominance of the meeting as PPI's primary modality contributes to the systematic exclusion of stroke survivors experiencing more severe stroke. This exclusion is not a failure of individual effort but reflects deeper issues about how involvement is imagined, resourced, and valued within research culture, and the place diversity holds within it.

### 4.1 The PPI Meeting as Infrastructure

The PPI meeting functions as infrastructure, in the sense developed by Star and Ruhleder [26], so embedded in practice that alternatives become unimaginable. It operates simultaneously as both a material practice and a discursive tool, shaping conventions within the community of practice of PPI practitioners. The meeting format has become so naturalised that it provides what appears to be a stable foundation for doing (and recording) PPI work. However, this very stability carries consequences. Like digital platforms, the meeting format encodes models of participation, including synchronous attendance, verbal communication, sustained concentration, and group comfort. Like all infrastructures, the PPI meeting is not neutral. It systematically advantages certain stroke survivors while excluding others from PPI activities. Yet the language of the ‘PPI meeting’ itself serves to obscure complexity, smoothing over innovation and difference. As Star and Ruhleder [26, p. 114] observe, ‘an infrastructure occurs when the tension between local and global is resolved’, but this resolution necessarily standardises practices, potentially erasing the very diversity that exists and inclusive PPI requires.

Our participants' accounts demonstrate innovation occurring largely *within* the language and framework of the PPI meeting, rather than questioning whether the meeting itself is fit for purpose. Even creative adaptations (one‐to‐one consultations, community outreach) were generally framed as supplementary to core meeting‐based work rather than legitimate alternatives.

This pattern mirrors findings across healthcare innovation more broadly. Cluley et al.'s [34] scoping review of 87 studies found that while PPI employed various methods, the group meeting remained the dominant organisational structure. Their review revealed PPI concentrated in earlier innovation stages (problem identification and invention) with limited evidence of involvement in adoption and diffusion, suggesting the meeting format may constrain not only whose participation is possible but also when in the innovation process involvement occurs.

This matters because, as Mosse [20] argues, formal participation structures can disembed lay knowledge from its social context. Knowledge that is embodied, relational, or expressed through non‐verbal means becomes lost when articulated through predetermined formats. For stroke survivors with severe communication difficulties, for example, the meeting format may not simply create access barriers. It may fundamentally constrain what knowledge can be shared and whose expertise counts.

### 4.2 The Politics of Resource Allocation

Resource constraints are important practical limitations. However, they can also be read as expressions of what is valued. The substantial, often invisible, labour required to complete PPI, from relationship‐building over time through to the coordination of participants, researchers, and the wider list of things needed across systems, frequently goes unfunded or inadequately resourced. When speech and language therapy support remains exceptional rather than standard in stroke PPI, despite stroke care requiring multidisciplinary teams, these are political choices about whose participation merits investment.

While these choices occur within constrained grant budgets, this framing obscures broader questions about the research culture's valuation of involvement work. The absence of reported long‐term funding for PPI outside of specific grant funding does not facilitate the ‘months, probably years’ of trust‐building needed, as one participant reports, to achieve diversity in PPI participants.

### 4.3 Proxy Participation

The widespread involvement of carers reveals complex questions about representation and voice. While some participants positioned carers as enabling stroke survivor participation, others suggested carers' perspectives were valued in their own right, and several groups included carers as members. This dual positioning of carers as supporting the involvement of those with more severe stroke and as stakeholders remained largely uninterrogated. When those with severe stroke are absent from PPI groups, does carer involvement constitute inclusion or inadvertently function as proxy representation?

Participatory design research demonstrates that adapted methods can enable people with cognitive and communication impairments to participate directly, negotiate across stakeholder groups and shape design decisions (see e.g. [35]). This raises critical questions about what PPI might learn from a more deliberate engagement with participatory design traditions—what would it mean to design involvement that enables those with severe stroke to participate directly, with carers in supporting rather than mediating roles? Our account echoes longstanding disability studies' critiques of proxy decision‐making and the medical model positioning carers as essential mediators of disabled people's voices [36]. Hamraie [37] demonstrates how infrastructures are designed around assumptions about ‘normal’ bodies and minds, rendering accommodation of other bodies an afterthought rather than a design principle. Reliance on carers, however well‐intentioned, may signal that direct participation by severely impacted stroke survivors is impossible, normalising exclusion rather than challenging the structures producing it.

Importantly, infrastructure designed to accommodate the most significant impacts of stroke would likely benefit diverse participation more broadly. Asynchronous methods, flexible timing, community‐based involvement and multiple participation modes would support not only those with severe stroke but also those in employment, with caring responsibilities, experiencing digital exclusion and for whom institutional spaces feel unwelcoming.

### 4.4 Individual‐Level Innovation Is Not Enough

Despite sophisticated understanding and creative adaptation, participants consistently acknowledged limited success in achieving diversity. Innovation occurred, but diversity did not follow, suggesting that individual‐level adaptations cannot overcome structural barriers. The very framing of adaptation positions severe stroke as exceptional, something requiring special accommodation within standard practice rather than central to design. Building involvement mechanisms accessible to those with the most significant impairments from the outset could benefit all participants rather than requiring constant adaptation. Our participants' accounts give a good starting point for thinking about what this might look like. They suggested offering multiple ways of contributing (email, text message or audio recordings, alongside or instead of live discussion), holding one‐to‐one conversations and home visits, involving stroke survivors through existing community and communication support groups where people already feel at ease, organising smaller groups matched by stroke severity, and including support from speech and language therapists and other specialists in PPI activity. The need for such redesign is particularly noteworthy given that participants also reported limited involvement of people from ethnic minority backgrounds despite deliberate efforts. Meetings privilege those with time flexibility, transport access, comfort in institutional spaces and confidence engaging in formal group settings. These requirements intersect with severe stroke to create compounded barriers for working‐age stroke survivors, those from minoritised ethnic communities, those who are generally frail, and those with limited prior research exposure.

Our findings illuminate how PPI infrastructure shapes participation. Russell et al.'s [24] critique of instrumentalisation in PPI, privileging measurable outputs over meaningful dialogue, is inferred throughout our participants' accounts. Our findings suggest the problem is not lacking frameworks but the taken‐for‐granted meeting infrastructure within which they often operate.

### 4.5 Limitations

Several limitations of this study merit acknowledgement. The questionnaire response rate (31 responses representing 104/185 eligible projects) means we cannot claim representativeness of stroke PPI practice nationally. Our aim was to understand the range of experiences across stroke PPI rather than measure prevalence, and the diversity of roles, institutions and project types represented supports analytical breadth. Those who did not respond may include teams with less active PPI programmes, suggesting our findings may present a more optimistic picture than the full population would warrant. Principal investigators were targeted because their details are publicly available; where they were not best placed to respond, they were asked to pass the invitation to the most suitable person in their team. Nonetheless, those with more direct knowledge of day‐to‐day PPI coordination, such as PPI leads, may not always have been the participant. As noted in the Results, and although acknowledged in other participants' accounts, the most detailed descriptions of the labour of doing PPI came from PPI lead responses, suggesting who responds may shape the granularity of findings. Relatedly, self‐report data on practices and barriers may reflect aspirational rather than routine conduct, particularly given the increasing scrutiny of PPI by funders, though our invitation framing and open‐ended questions sought to mitigate this. Our sample also covered projects funded through NIHR, UKRI and the Stroke Association, meaning practices outside of formal funding were not captured. Finally, while infrastructure provides a productive analytic lens for understanding systemic patterns, it does not fully account for the agency of individual practitioners working creatively within constraining structures, an important tension that subsequent qualitative work, currently underway, will explore in greater depth.

### 4.6 Implications for Practice

Inclusive stroke PPI would require fundamental shifts in funding, infrastructure, and practice. This means valuing and developing expertise and skills in PPI, protecting time for relationship‐building as a routine part of PPI and research work, and including professional support (SLT, occupational therapy, neuropsychology) as standard in stroke research budgets. It requires long‐term funding for PPI that exists outside of individual project grants, enabling sustained community engagement beyond project timescales. It also requires funders and research teams to recognise diverse involvement methods (e.g., asynchronous contribution, one‐to‐one consultation, community‐based deliberation) as legitimate approaches to PPI in their own right rather than add‐ons to meetings.

## 5 Conclusion

Stroke PPI offers an important case to examine whose voices matter in health research. The problem is not individual but structural: taken‐for‐granted formats, inadequate resources, and the absence of specialist support, dedicated coordination and sustained funding. The solutions must therefore also be structural, moving from adapting meetings to designing involvement around those with the most significant impairments from the outset. Until these issues are addressed, PPI risks amplifying certain voices while claiming to represent patients and the public more broadly, a claim we should find troubling given health research's stakes for those most affected by health conditions.

## Author Contributions

**David Wyatt:** conceptualisation, funding acquisition, methodology, data analysis, writing – original draft preparation, writing – reviewing and editing. **Sophie Rowland‐Coomber:** conceptualisation, funding acquisition; methodology, data analysis, writing – reviewing and editing. **Eleanor Stevens:** conceptualisation, methodology, data analysis, writing – reviewing and editing. **Christopher McKevitt:** conceptualisation, funding acquisition, data analysis, writing – reviewing and editing. **Madeline Cruice:** conceptualisation, funding acquisition, data analysis, writing – reviewing and editing. **Timothy Neate:** conceptualisation, funding acquisition, data analysis, writing – reviewing and editing. **Eva S. Emmett:** data analysis, writing – reviewing and editing. **Matthew D. L. O'Connell:** funding acquisition, data analysis, writing – reviewing and editing. **Charles Wolfe:** conceptualisation, funding acquisition, data analysis, writing – reviewing and editing. **Iain J. Marshall:** conceptualisation, funding acquisition, data analysis, writing – reviewing and editing.

## Ethics Statement

The study received ethical approval from King's College London Research Ethics Committee reference LRS/DP‐23/24‐42186.

## Conflicts of Interest

The authors declare no conflicts of interest.

## Supporting information


Supporting File

## Data Availability

There is no permission from the participants to share the data.
