# Supplementary material for: Beyond the Meeting: Patient and Public Involvement Practices and Barriers to Inclusive Participation in Stroke Research
Source: Health Expect. 2026 Oct 5;29(5):e70903. doi: 10.1111/hex.70903 (PMC13639546; doi:10.1111/hex.70903)
Supplement: Supplementary file 1 [file HEX-29-e70903-s001.pdf]

Equitable and Inclusive Patient and Public Involvement in Stroke Research

Ethical Approval: LRS/DP-23/24-42186

This form is part of a wider National Institute for Health and Care Research (NIHR) funded project that explores how we can support the involvement of those with complex stroke-related impairments in patient and public involvement (PPI) activities. As you will know, stroke survivors face major barriers to participating in PPI and research. The impacts of stroke are far-reaching, affecting survivors' communication, cognition, vision, movement, social participation and mental health. Current PPI, often in the form of PPI meetings, can sometimes facilitate the involvement of some stroke survivors, but not all, particularly excluding those with complex impairments.

This form is part of work to scope existing knowledge and practices in stroke PPI. This scoping work will later inform

the co-development of evidence-based and innovative methods of completing PPI that are inclusive of different stroke-related impairments and share this new knowledge widely to (stroke) research, stroke survivor and PPI communities.

Why have I been invited to take part?

You are being invited to participate in this project because you complete PPI activities with stroke survivors.

This form has been distributed by email to researchers working in the field of stroke who were funded by the National Institute for Health and Care Research (NIHR), UK Research and Innovation (UKRI) and the Stroke Association for projects that were active on or after 1 October 2019. You may have been forwarded the email linking this form by a colleague because you are best placed to respond to the questions asked.

What will happen if I take part?

Participation is completely voluntary and responses will be used anonymously. Should you choose to complete this form, you will be asked for information on the stroke PPI work you are involved in. The form asks a little about how your PPI work takes place, who attends and how it is organised. It also asks you to share any documents you use in (for example, terms of reference), or are produced

by (for example, publicly available minutes), your PPI work.

If you have any documents to hand, this form should take no longer than 15 minutes to complete.

You may also be invited, at a later date, to complete a semi-structured interview on your experiences and practices of involving stroke survivors in research.

Further information about the project can be found on the Participant Information Sheet linked here. Should you have any further questions about this project, please contact david.wyatt@kcl.ac.uk.

Thank you for considering participating in this study.

FUNDED BY
NIHR | National Institute for Health and Care Research

Digital Consent Form

You will only be able to continue if you answer yes to all of the following questions.

I confirm that I have read and understood the information sheet linked on previous page and **here** dated 31/10/2024 WP1 Version 2.0. I have had the opportunity to consider the information and ask questions, which have been answered to my satisfaction.

- ○ Yes

I consent voluntarily to be a participant in this project and understand that I can refuse to take part and can withdraw from the project at any time, without having to give a reason, up until 31 March 2025.

- ○ Yes

I understand my personal information will be processed for the purposes explained to me in the Information Sheet. I understand that such information will be handled under the terms of UK data protection law, including the UK General Data Protection Regulation (UK GDPR) and the Data Protection Act 2018.

- ○ Yes

I understand that my information may be subject to review by responsible individuals from the College for monitoring and audit purposes.

- Yes

I understand that confidentiality and anonymity will be maintained, and it will not be possible to identify me or the organisation(s) I work for in any research outputs.

- Yes

I understand that the information I have submitted will be published as academic papers, blog entries, conference posters and or conference presentations.

- Yes

These first questions ask a little about you (for example, your name, institution, email address) and some basic details about the funded, stroke research projects you have been involved in. We are particularly interested in those that were active either on or after 1 October 2019.

What is your name?

What is your job title?

What is your email address?

At what institution(s) are you employed?

Please list the stroke research projects you are involved in that were active on or after 1 October 2019.

We have publicly available information on what stroke research projects have been funded during this time. This information however is limited, and often only including the name of the Principal Investigator, and not the numerous other people involved in delivering research (and stroke PPI in particular).

	Information on stroke research			
	Title of research project	Project funder	Chief Investigator/Principal Investigator	Grant type - e.g. project, programme, fellowship, studentship
Project 1				
Project 2				
Project 3				
Project 4				

If you need further space to list your projects, please use this box.

A key part of this project is understanding current practices in stroke PPI.

In the box below, please provide brief information about your stroke PPI group(s). Please include details on:

- how many people you involve in your work
- the basic demographics of your participants
- how you organise your involvement work (for example, what format it takes, whether this is face-to-face or online)
- how researchers engage with your PPI participants (for example, what kinds of input are sought?)
-

We are interested in understanding the resources involved in arranging and completing PPI activities. In the box below please could you break down for your most typical PPI activity:

1. the time spent completing different parts of arranging this PPI activity. This might include the amount of time spent inviting and communicating with participants, arranging rooms/refreshments, communicating with those who will discuss research with the group and other activities.
2. any financial outlay (beyond staff time covered above). This might include, paying PPI participants, hiring rooms, refreshments and other activities.

Please provide details below of anything you do to encourage and support the presence and involvement of those with more severe stroke-related impairments.

How can we make stroke PPI more inclusive of all stroke survivors, irrespective of stroke-related impairments?

Please upload here any documents you feel comfortable sharing that are used in, or generated by, your patient and public involvement activity.

While you are free to upload anything you deem relevant, this might include:

- any terms of reference used by your stroke PPI group(s),

- any published resources (like academic papers, grey literature) that are useful in your PPI activities, and
- any public minutes or other records of your PPI activities.

Please note, this form only accepts one file upload per question. You can either compress your files into one zip file and upload one file or upload one of your files here and answer 'yes' to the next question where you will be able upload files individually.

There is space later in this form to add any weblinks to resources or tools used in your PPI work.

Do you have any additional files you would like to share?

- ○ No
- ○ Yes

Please upload a file here:

Please upload a file here:

Please upload a file here:

Please upload a file here:

Please upload a file here:

Please upload a file here:

Please upload a file here:

Please upload a file here:

Please upload a file here:

Please upload a file here:

If there are any documents or resources that it's easier to share via a weblink, please link here:

Are you happy to be contacted at a later date about this project?

- No
- Yes

Would you like to receive information about the informal network of those working in stroke PPI we will convene as part of this project?

- No
- Yes

Powered by Qualtrics
